# The first assessment to detect *Mycoplasma hyopneumoniae* by sampling laryngeal swabs to investigate sow stability in South Korea

**DOI:** 10.1186/s12917-020-02663-2

**Published:** 2020-11-23

**Authors:** YuSik Oh, JongHyuk Baek, JoongBok Lee, Sun-Hee Cho, Changhoon Park

**Affiliations:** 1 Boehringer Ingelheim Animal Health Korea Ltd., Seoul, South Korea; 2 Department of Infectious Diseases, College of Veterinary Medicine, Konkuk University, Seoul, South Korea; 3 Department of Animal Vaccine Development, BioPOA, 593-26 Dongtangiheung-ro, Hwaseong-si, Gyeonggi-do Republic of Korea; 4 Department of Microbiology and Immunology, Eulji University School of Medicine, Yongdu-dong, Jung-gu, Daejeon, South Korea

**Keywords:** *Mycoplasma hyopneumoniae*, Laryngeal swab, Vertical transmission, Herd size, Acclimation

## Abstract

**Background:**

*Mycoplasma hyopneumoniae (M. hyopneumoniae)*, a representative pathogen causing swine enzootic pneumonia, generally infects piglets vertically. However, it is difficult to ascertain the *M. hyopneumoniae* infection state of sows due to limited detection methods. This report investigated sow herd stability by applying nested PCR to laryngeal swabs of suckling pigs, which is reportedly the most sensitive method.

**Results:**

*M. hyopneumoniae* was detected in 14 farms (63.6%) and 127 piglets (6.5%). The prevalence of sows likely to transmit *M. hyopneumoniae* in herds (11.1%) was calculated. In addition, there was a significant difference in detection rates among farms depending on herd size, gilt replacement rate, acclimation method, and antibiotic usage, suggesting various parameters that influence sow stability.

**Conclusions:**

The results demonstrated that laryngeal swabs from suckling pigs have provided useful information regarding vertical transmission from sows in South Korean farm conditions. This result demonstrated that farms with larger herd sizes, higher gilt replacement rates, and a practice of naturally exposing gilts for acclimation had higher detection rates in weaning piglets, indicating an unstable sow infection state.

## Background

Swine enzootic pneumonia (EP), a chronic respiratory disease in pigs of all ages characterized by dry coughing, growth retardation, and poor feeding efficiency, is mainly caused by *M. hyopneumoniae* [1]. *M. hyopneumoniae* adheres to the ciliated epithelium of the respiratory tract of the pig. After attachment, the infected pigs show chronic coughing and increased susceptibility to other respiratory infections. Due to these symptoms, the pigs have reduced weight gain [2]. In some cases, other species of *Mycoplasma*, such as *M. hyorhinis* or *M. hyosynoviae*, are also able to infect pigs. *M. hyorhinis* commonly causes polyserositis and arthritis in nursery pigs. *M. hyosynoviae* causes arthritis in grower-finishers [3]. However, the tremendous economic loss in the swine industry was caused by *M. hyopneumoniae* due to decreased performance, increased treatment and vaccination costs [4].

Swine enzootic pneumonia caused by infection with *M. hyopneumoniae* is one of the most common diseases on pig farms worldwide. Several abattoir surveys have demonstrated that a high prevalence of enzootic pneumonia is associated with lung lesions in pigs [5–7]. In South Korea, antibodies against *M. hyopneumoniae* were mainly detected in pigs 14–22 weeks old [8]. In another report, 67.8% of the collected oral fluid samples and 87.5% of the investigated farms were polymerase chain reaction (PCR) positive in the country [9].

Vertical transmission of *M. hyopneumoniae* from an infected sow to her piglets is recognized as the typical pathway of enzootic pneumonia. Infected sows are considered a risk factor for *M. hyopneumoniae* infection in the preweaning period [10]. *M. hyopneumoniae* shedding through direct contact with vertically infected piglets is the initial infection source in susceptible pig populations during the nursery and growth-finishing periods [10]. “Sow herd stability” has been described as an absence of clinical signs and no evidence of pathogen circulation within the sow herd. Generally, sow herd stability is thought to be directly linked to the entire herd stability due to vertical transmission. The proportion of positive piglets in each weaning group was correlated with the proportion of positive sows in the group. Therefore, in terms of sow stability, the source of gilts, herd size, stocking density, and ventilation system are the main important risk factors for enzootic pneumonia linked to *M. hyopneumoniae* [11–13].

For conclusive diagnosis, culturing of *M. hyopneumoniae* has been considered the gold standard. However, it is not used for routine diagnosis due to the highly time-consuming process. The organism can also be detected by immunofluorescence testing, but the test has limited sensitivity. Although serology can be used to show the presence of *M. hyopneumoniae* at a herd level, it is not proper for diagnosis on individual animals [14]. Currently, nested PCR testing has been demonstrated as the most sensitive tool to detect extremely low levels of nucleic acids [15].

A recent study indicated that laryngeal swabs investigated with the PCR method are superior in detecting early *M. hyopneumoniae* infection compared to the more commonly used investigation of nasal swabs and tracheobronchial lavage fluid in eight-week-old pigs [16]. PCR results for laryngeal swab samples had the highest sensitivity of all sample types at 5 days after infection, with a detection rate of 81%. The present study aimed to investigate the sow herd stability of *M. hyopneumoniae* using laryngeal swabs of suckling pigs. Although *M. hyopneumoniae* is barely detected in adult pigs, the detection rate of weaning pigs that were vertically infected by sows was comparably high. This is the first report of infection and virus shedding among sows in South Korean field conditions, and it was conducted by testing the laryngeal samples of weaning piglets with nested PCR.

## Methods

### Farms

Twenty-two pig farms in South Korea were selected for testing. To investigate the test, swine veterinarians visited 22 farms under the contract. Without consideration of specific geological area, the veterinarians selected 22 farms according to pig population from four representative swine producing provinces of South Korea (three farms in Gyeonggi-do (19% of the total national pig population); eight farms in Chungcheong-do (28%); six farms in Jeolla-do (25%); five farms in Gyeongsang-do (23%)). Accordingly, the farms were distributed in balance across the nation.

The farms had a higher number of marketed pigs per sow per year (MSY) than the average South Korean pig farm and were managed with a three-way cross breeding program (Duroc, Landrace, Yorkshire). The average MSY in South Korea was reportedly 17.9 in 2018 based on the Korean pork producer association. The average MSY of 22 farms joined in this study was 21.8. Only three farms (no. 15, 16, and 17) had less MSY than 20. Farm no.15 and 17 had unexpected events including porcine reproductive and respiratory syndrome (PRRS) and porcine epidemic diarrhea (PED) outbreak a year before the experiment. Farm no.16 had a sudden change of gilt source. Their relatively low MSY might result from such internal factors.

All farms had farrow-to-finish production systems and weaned their piglets at three to four weeks of age, while each farm had individual acclimation, antibiotics, and gilt replacement protocols (Table 1). The exposure method to naturally expose the gilts to *M. hyopneumoniae* for acclimation was selected on three farms, and vaccination with commercial products (Ingelvac^®^ MycoFLEX and Zoetis Respisure ONE^®^) was used on 14 farms. While investigating, antibiotics (amoxicillin, enrofloxacin, tilmicosin, ceftiofur, penicillin, streptomycin, gentamicin, tulathromycin, lincomycin, and spectinomycin) were used. In the case of antibiotics, there were three categories associated with the treatment. The sows and growing pigs were inoculated with antibiotics on randomly selected farms: only growing pigs on seven farms, only sows on five farms, and both on six farms. In the case of gilt introduction, seven farms introduced gilts from their own female pigs, and 15 farms brought gilts from grandparent farms (GP). Their protocols are usually used in South Korean swine production systems.

**Table 1:** Overview of farm management and *M. hyopneumoniae* detection in piglets

Farm no.	MSY^a^	Herd size^b^	Gilt replication rate	*M. hyo* status of gilt^c^	Gilt source	Acclimation	Antibiotics (piglet treatment)	Antibiotics (Sow treatment)	PRRSV detection^d^	*M. hyo* prevalence^e^
										Pos/total
1	21	≤ 550	≤ 40%	pos	Self-replacement	Ingelvac MycoFLEX^®^			pos	7/90
2	20	≤ 550	≤ 40%	pos	Self-replacement	Ingelvac MycoFLEX^®^	Ceftiofur		pos	0/90
3	24	≤ 550	> 40%	pos	Self-replacement	Respisure-ONE^®^	penicillin + Streptomycin		neg	0/90
4	20	> 550	≤ 40%	pos	Self-replacement	Respisure-ONE^®^	Ceftiofur		pos	9/90
5	24	> 550	≤ 40%	neg	External (GP)	Ingelvac MycoFLEX^®^	Gentamicin		pos	0/90
6	22	> 550	≤ 40%	pos	External (GP)	Ingelvac MycoFLEX^®^			pos	2/90
7	26.5	> 550	> 40%	pos	External (GP)	Respisure-ONE®	Tulathromycin		neg	3/88
8	23	> 550	> 40%	neg	External (GP)	Exposure	Ceftiofur	Amoxicillin	pos	6/90
9	25	≤ 550	> 40%	neg	External (GP)	None			neg	0/90
10	22	≤ 550	≤ 40%	neg	External (GP)	Ingelvac MycoFLEX^®^	Tulathromycin	Tiamulin	pos	5/90
11	20	≤ 550	≤ 40%	pos	Self-replacement	Ingelvac MycoFLEX^®^		Tilmicosin	pos	0/90
12	21	> 550	> 40%	neg	External (GP)	Ingelvac MycoFLEX^®^		Tilmicosin	pos	0/90
13	23.5	> 550	> 40%	pos	External (GP)	Respisure-ONE^®^	Lincomycin+ Spectinomycin	Amoxicillin	neg	26/90
14	24.2	≤ 550	> 40%	pos	Self-replacement	Exposure	Lincomycin+ Spectinomycin		neg	24/90
15	18	≤ 550	≤ 40%	pos	External (GP)	Ingelvac MycoFLEX^®^	Lincomycin+ Spectinomycin		pos	0/66
16	16.5	> 550	≤ 40%	pos	External (GP)	Ingelvac MycoFLEX^®^	Tulathromycin	Lincomycin+ Spectinomycin	pos	2/88
17	17	> 550	≤ 40%	pos	Self-replacement	None		Amoxicillin	pos	3/90
18	20	> 550	≤ 40%	neg	External (GP)	Ingelvac MycoFLEX^®^		Amoxicillin	pos	13/90
19	22.5	> 550	> 40%	neg	External (GP)	Exposure	Amoxicillin	Lincomycin + Tiamulin	pos	15/90
20	20.3	≤ 550	≤ 40%	pos	External (GP)	None		Penicillin	pos	0/90
21	24.3	≤ 550	> 40%	neg	External (GP)	None	Enrofloxacin or Sulfamethoxazole+ Trimethoprim	Amoxicillin	pos	11/90
22	25	≤ 550	≤ 40%	neg	External (GP)	None	Ampicillin		neg	1/90

^a^Number of pigs, which survive until they reach to the weight for sale and are sold, among pigs produced by a sow for a year

^b^Numbers of sows on individual farms

^c^Anti–*M. hyopneumoniae* antibody detection results from a previous test

^d^PRRSV antigen test (PCR) of weanling piglet serum

^e^Number of positive piglets/total number of piglets tested by nested PCR for *M. hyopneumoniae* on the three sampling dates

*pos* positive, *neg* negative

The veterinarians reported signs of porcine respiratory disease complex (PRDC) in growing pigs based on previous tests, which were performed to monitor the herd disease state at least 1 month prior to this experiment. The antibodies against *M. hyopneumoniae* from randomly collected sera were detected by ELISA (BioCheck), and PCR results of necropsied lung tissue showed the presence of *M. hyopneumoniae*. Including *M. hyopneumoniae,* type 1 and type 2 PRRSV, porcine circovirus type 2d (PCV2d) and bacteria (*Salmonella, Streptococcus suis*) were also isolated from some cases. Concretely, 6 farms reported the presence of type 1 PRRSV, and 7 farms reported type 2 PRRSV. Only 3 farms had two PRRSVs simultaneously. PCV2d was the only porcine circovirus type on the farms and was detected on 5 farms. *Salmonella* and *Streptococcus* were isolated from one farm.

### Laryngeal swab sampling

Sampling was conducted as previously described [16]. Briefly, a BD CultureSwab™ Liquid Stuart Single Swab (BD Diagnostics, Sparks, USA) was inserted by a veterinarian right behind the epiglottis of a piglet using a laryngoscope. Two- to three-week-old pigs were randomly taken for sampling from at least 15 sows of one farrowing batch, and sampling was performed thrice on each of the 22 farms. The sample size was determined based on the statistical 95% confidence limit. For the previous data, the detection limit of the positive sample number in the 1000 population was 29. Consequently, 30 samples were needed from each herd.

In most farms, there were 15 ~ 20 farrowing sows in each batch, and most of them were selected for sampling. There were newborn piglets every week because all farms had the same weekly production system. The test was conducted 1 day per week in three consecutive weeks, and the date was selected by veterinarians during October and November 2018.

### *M. hyopneumoniae* DNA extraction & nested PCR

Laryngeal swab samples were processed for DNA isolation with a Viral gene spin RNA/DNA extraction kit (iNtRon Biotechnology, South Korea) on the sampling day, and nested PCR was performed with isolated DNA as previously described [15]. The positive control (*M. hyopneumoniae* strain SNU98703 culture supernatant) was tested for nested PCR sensitivity. The reaction detected *M. hyopneumoniae* DNA to a dilution of 10^− 8^, approximately 1 fg, as in a previous report [17]. A pristine swab in DNase and RNase-free water (Sigma-Aldrich, Stockholm, Sweden) was used as the negative control. All nested PCRs were duplicated to confirm the responses.

### Statistical analysis

The chi-square test and Fisher’s exact test were used to compare the prevalence of infected piglets with the following parameters: herd size, gilt replacement rate, acclimation procedures, and antibiotic usage. A value of *P* < 0.05 was considered significant.

## Results

### Detection of *M. hyopneumoniae* DNA

*M. hyopneumoniae* DNA was detected by nested PCR in 14 farms (63.6%) and 127 piglets (average 6.5%, range 0–28.8%). After sequencing PCR products, 16S ribosomal RNA of *M. hyopneumoniae* was confirmed. The prevalence of sows likely to transmit *M. hyopneumoniae* (11.1%) was calculated as the ratio, specifically, the number of sows with *M. hyopneumoniae*-positive piglets divided by the number of total sows. The mean prevalence of 13% at the first sampling time was reduced to 2.2% at the final sampling time.

### Prevalence of positive piglets with regard to herd size

**Fig. 1:**
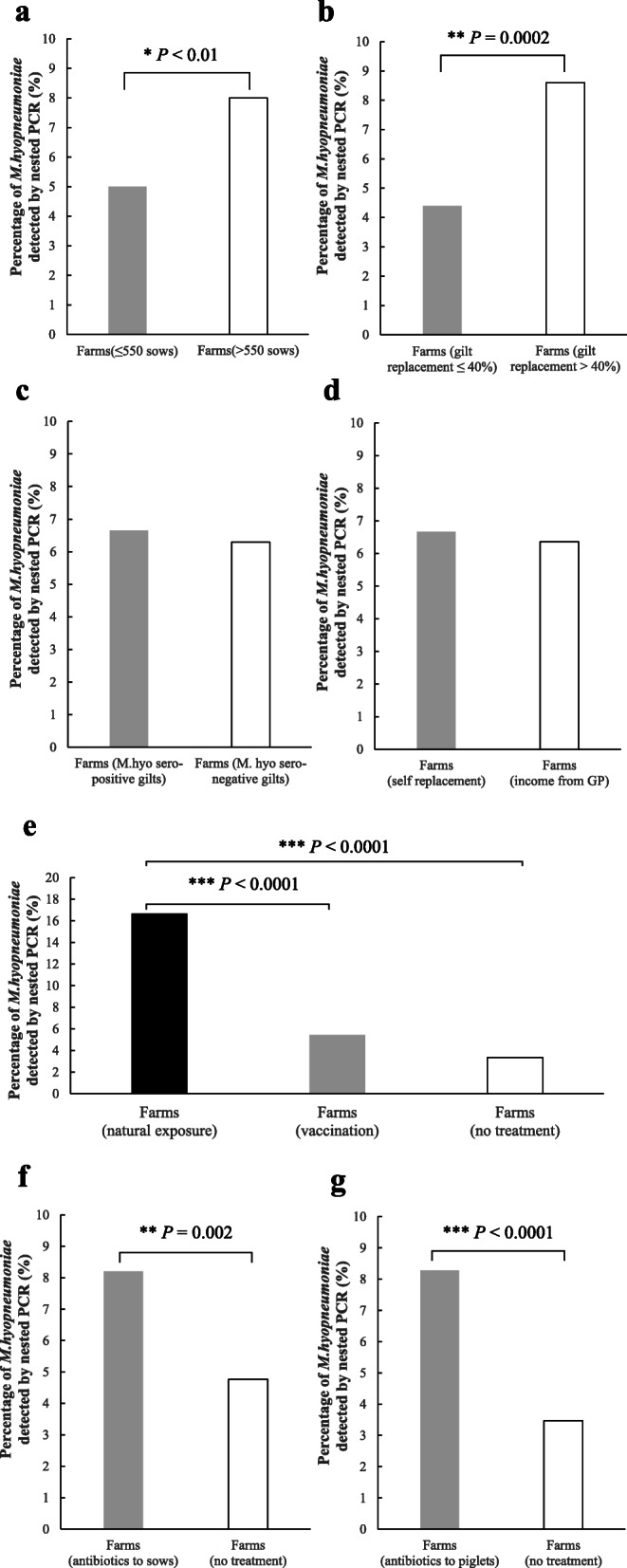
Comparison of the prevalence of *M. hyopneumoniae* between groups. **a** Comparison of detection rates by herd size: positive piglet prevalence in farms with > 550 sows (□) and others (■). **b** Comparison of detection rates by gilt replacement rate: prevalence of positive piglets in farms with > 40% replacement (□) and others (■). **c** Comparison of detection rates by gilt status: prevalence of positive piglets in farms introducing serologically positive (■) and negative gilts (□). **d** Comparison of detection rates by gilt source: prevalence of positive piglets in farms that produce their own replacement gilts (■) and farms that acquire their gilts from GP (□). **e** Comparison of detection rates by acclimation method: prevalence of positive piglets in farms that naturally expose gilts to *M. hyopneumoniae* (■), farms that vaccinate gilts (■), and farms that do not use acclimation treatment (□). **f** Comparison of detection rates by antibiotic usage for sows: prevalence of positive piglets in farms that use antibiotics on sows (■) and farms that do not use such treatment (□). **g** Comparison of detection rates by antibiotic use on piglets; positive piglet prevalence in farms that use antibiotics on piglets (■) and farms that do not use such treatment (□)

*M. hyopneumoniae* DNA was detected in 48 piglets (5%) from 11 farms with ≤550 sows and 79 piglets (8%) from 11 farms with > 550 sows. Farms with > 550 sows had a significantly higher detection rate (*P value* < 0.01) (Fig. 1a).

### Prevalence of positive piglets with regard to the replacement rate of gilts

*M. hyopneumoniae* DNA was detected in 42 piglets (4.4%) from 13 farms that replaced ≤40% of gilts and 85 piglets (8.6%) of 9 farms replacing > 40%. Farms replacing > 40% of gilts had a significantly higher detection rate (*P value* = 0.0002) (Fig. 1b).

### Prevalence of positive piglets with regard to gilt source

*M. hyopneumoniae* DNA was detected in 76 piglets (6.7%) from 13 farms introducing serologically *M. hyopneumoniae*-positive gilts and 51 piglets (6.3%) from nine farms introducing serologically *M. hyopneumoniae*-negative gilts (Fig. 1c). *M. hyopneumoniae* was detected in 43 piglets (6.8%) from seven farms with self-replacement of gilts and in 84 piglets (6.4%) from 15 farms with gilts brought from grandparent farms (Fig. 1d). There was no significant difference among these farms.

### Prevalence of positive piglets with regard to gilt acclimation

*M. hyopneumoniae* DNA was detected in 45 piglets (16.7%) from three farms that naturally exposed gilts to *M. hyopneumoniae*. *M. hyopneumoniae* DNA was detected in 67 piglets (5.4%) from 14 farms that vaccinated gilts. Finally, *M. hyopneumoniae* DNA was detected in 15 piglets (3.3%) from five farms without acclimation treatment. Farms that naturally exposed gilts to *M. hyopneumoniae* had a significantly higher detection rate than the others (*P value* < 0.0001) (Fig. 1e).

### Prevalence of positive piglets with regard to usage of antibiotics

*M. hyopneumoniae* DNA was detected in 81 piglets (8.2%) from 11 farms using antibiotics in the sow herds and 46 piglets (4.7%) from 11 farms that did not (Fig. 1f). The pathogen was detected in 102 piglets (8.3%) from 13 farms using antibiotics in suckling pigs and 25 piglets (3.4%) from nine farms that did not. The results showed that the detection rate was significantly higher in farms that used antibiotics in sows or piglets (*P value* < 0.002) (Fig. 1g).

## Discussion

*M. hyopneumoniae* is a main causative pathogen of porcine respiratory disease complex (PRDC) [1]. PRDC is characterized clinically by anorexia, slow growth, lethargy, fever, dyspnea and an antibiotic treatment-resistant cough in growing and finishing pigs [18, 19]. To prevent PRDC, detection of *M. hyopneumoniae* during the early stages of infection is critical but technically challenging. The colonization of pigs by *M. hyopneumoniae* starts from tracheal cilia as the initial postexposure infection site. Therefore, direct access into the trachea cilia could be useful for sensitive detection of *M. hyopneumoniae.* And the tracheobronchial lavage fluid may be regarded as the best sample to detect the infected microorganism in this case [20]. However, in previous reports, laryngeal swabs have demonstrated the highest sensitivity for *M. hyopneumoniae* DNA detection among oral, nasal, and tracheobronchial samples as well as antibody detection in serum samples [16, 21, 22]. Although the larynx is not considered the typical location for *M. hyopneumoniae* infection or colonization, it may function as a vestibular site that may incur lower airways. Consequently, the larynx could be the place where *M. hyopneumoniae* is concentrated, and it is a preferred sampling site for favorable access and proximity to the lower airways [16, 23]. As a field study, our results show that laryngeal swabs for *M. hyopneumoniae* detection are practical and reliable.

As a result, *M. hyopneumoniae* DNA was detected by nested PCR in 14 farms (63.6%) and 127 piglets (average 6.5%, range 0–28.8%). The prevalence (11.1%) of sows likely to transmit *M. hyopneumoniae* in herds was calculated as the ratio of total sows to sows with pathogen-positive piglets. Compared to previous reports, it was similar to the results obtained in other countries. Using laryngeal swabs, 7% of weanling pigs and more than 50% of sow herds tested positive for *M. hyopneumoniae* in the USA. In Germany, 18.7% of weanling pigs and 75% of sow herds were PCR positive [24].

There was a significant difference depending on herd size and gilt replacement rate. Farms with more than 550 sows had a significantly higher detection rate than those with lower sow numbers. Farms where more than 40% gilts were replaced also had a significantly higher detection rate than those where gilts were less replaced. In general, larger farms tend to replace gilts at a higher rate. Larger farms or high gilt replacement rates may support the transmission of pathogens due to management factors such as biosecurity, vaccination practices, and herd density [25]. However, larger farms may have additional factors, such as cross fostering in the farrowing unit, contamination by teeth grinding, tail docking, castration and housing, that have not been investigated in this survey, influencing the results.

There was no significant difference in the prevalence of *M. hyopneumoniae* detection in piglets among farms with regard to the serological status of introduced gilts. Similarly, the detection rate in farms with self-replacing gilts was not significantly different from the rate in farms with gilts brought from grandparent farms. With regard to the long shedding period, sows exposed to *M. hyopneumoniae* at the age of 50–100 days are expected to develop immunity early enough so that the possibility of transmission is reduced at their first farrowing [26].

Notably, farms where gilts were naturally exposed to *M. hyopneumoniae* had a significantly higher detection rate in piglets than farms with vaccinations or without acclimation. Considering that experimentally infected pigs shed *M. hyopneumoniae* for up to 200 days [27], natural exposure for gilt acclimation at an age of 100 days or older may support vertical transmission. According to the gilt acclimation survey of 22 farms, no farms had diagnostic verification after gilt acclimation. Seventeen farms introduced gilts over 140 days old, and 16 farms had acclimation periods below 10 weeks. Inappropriate acclimation resulted in a high risk of sow stability.

Amoxicillin, enrofloxacin, tilmicosin, ceftiofur, penicillin, streptomycin, gentamicin, tulathromycin, lincomycin, and spectinomycin were used on most farms. Treatments were applied to sows, piglets, or both. Interestingly, the detection rate was significantly higher in farms that used antibiotics in the sow herds than in those that did not use antibiotics. The detection rate was also significantly higher in farms using antibiotics in suckling pigs than in farms where antibiotics were not used. Although antibiotics have been demonstrated to be effective at controlling enzootic pneumonia [28], only limited information is available with regard to the reduction of transmission. It may be interpreted that the farms were facing serious PRDC problems when there was a higher detection rate of *M. hyopneumoniae* in the farms where antibiotics were actively used. Farms with severe enzootic pneumonia outbreaks tend to use antibiotics more frequently as a control measure. In addition, the symptoms may reappear after cessation of treatment or development of antimicrobial resistance [2].

As a result of subpopulation in a batch, there was a great deal of variability in the prevalence between farrowing batches within a farm. The mean prevalence at the first sampling time was 13%. In the second sampling time, a mean prevalence of 4.9% was reported. For the third sampling time, the mean prevalence was 2.2%. These results suggest that the time of infection and shedding of *M. hyopneumoniae* is not consistent in the different farrowing batches. In addition, a minimum of three sampling time points is necessary to assess sow herd stability. The necessity of multiple samplings has already been suggested [29].

## Conclusions

In summary, this study investigated the difference in *M. hyopneumoniae* prevalence in weaning-age or approximately weaning-age piglets among farms. The results demonstrated that laryngeal swabs of suckling pigs provided useful information with regard to sow herd stability in farms with enzootic pneumonia. In addition, it provided some insight into influencing parameters such as herd size, gilt replacement, acclimation, and antibiotic usage.

## Data Availability

The dataset collected in the current study is available from the corresponding author upon reasonable request.

## Abbreviations

*M. hyopneumoniae*: *Mycoplasma hyopneumoniae*
PCR: Polymerase chain reaction
MSY: Marketed pigs per sow per year
PRDC: Porcine respiratory disease complex
